# Research on the insurance of swimming crab temperature and salinity index insurance based on Copula function

**DOI:** 10.1371/journal.pone.0272940

**Published:** 2022-08-15

**Authors:** Xiaofang Shi, Mingjie Zhao, Yu Xu, Yanjuan Wu, Xiaolin Sun, Ke Jin, Bo Qiu, Chao Gao

**Affiliations:** 1 Department of Geography and Spatial Information Techniques, Ningbo University, Ningbo, China; 2 Business School of Ningbo Universities, Ningbo Universities, Ningbo, China; 3 Ningbo Universities Collaborative Innovation Center for Land and Marine Spatial Utilization and Governance Research at Ningbo University, Ningbo University, Ningbo, China; ADYÜ: Adiyaman Universitesi, TURKEY

## Abstract

Under climate change, the sea surface temperature and salinity change greatly, which poses a considerable threat to sustainable food security. Sea surface temperature and salinity (SST/SSS) are selected to examine the annual output of swimming crab in 24 cities along the eastern China. The Copula-based function was used to construct the probability distribution model of the swimming crab yield with SST and SSS. The pure premium rate of the swimming crab production in these 24 cities are also examined. The results show that 1) There is significant positive correlations between the yield of swimming crab with temperature and salinity over the study area. The only exception is that the correlation between yield of swimming crab and salinity is not significant in the south of study area. 2) The span of the pure insurance premium rate of swimming crab in 24 cities increases rapidly with the increase of the protection level, the maximum span up to 2.04%, and the minimum span is only 1.6%. 3) The distribution of the swimming crab insurance premium rate is various in space. The insurance premium rate of 8 cities in the south of Taizhou is low with the highest premium rate at 5.6%. The insurance premium rate of 16 cities in north of Taizhou is relatively high with the rate between 6%-22%. The research can provide a theoretical basis for the pricing of insurance products for swimming crab in 24 cities in the typical aquaculture areas in eastern China.

## 1 Introduction

Under climate change, extreme weather events are likely to become more frequent and intense, which have a great impact on mariculture [1]. The direct economic loss caused by meteorological disasters to China’s mariculture industry is as high as 15.637 billion yuan in 2019. Insurance products compensated for losses which played a particularly prominent role in compensation for aquaculture losses [2, 3]. The swimming crab with rich nutrition and delicious taste is an important aquaculture species in China, which produce large economic benefits. It has been listed as a key aquaculture product in China in 1981 [4]. The yield of swimming crab is affected by natural factors, breeding technology, and labor, etc. Sea surface temperature and salinity (SST/SSS) are the most important environmental factors affecting the growth conditions and development of swimming crab, including the survival rate, production, breeding cost, pests and diseases. The resulting losses are often high and unbearable for smallholders. Therefore, we choose SST and SSS as variables to determine swimming crab premium rates, which can theoretically improve the accuracy of the rate results. There was a certain practical significance to investigate the swimming crab index insurance.

Index insurance was proposed to link the uncertainty of crop yield with meteorological disasters as early as 1999 [5]. The first application of index insurance in the Chinese aquaculture industry is the Neitang crab hydrological index insurance product, which was developed by the People’s Insurance Company of China (PICC) Jiangsu Branch in 2012 [6]. Currently, there are several major studies on index insurance in the following aspects: 1) In terms of spatial scale, the relevant researches mainly select the national, provincial and municipal region as the research area. Some studies determine the insurance rate by establishing the relationship between the production of aquaculture products and natural factors, and some studies compare the differences of index insurance products in different study areas. [7–10]. The current study lacks a particular study of the insurance rates in a single aquacultural product in the entire eastern coastal area of China. 2) In terms of data source, index insurance researches mainly designed based on data of observed wind [11], temperature [12], precipitation [13, 14], sunshine duration [15] and other data monitored by meteorological stations. Previous theoretical researches are continuously applied to practice. The current studies mainly investigate the relationship between farmed product and single factors. However, the growth status of aquaculture products is jointly affected by multiple meteorological and hydrological factors, and the changes in SST and SSS will directly affect the yield of aquaculture products. In view of this, SST and SSS as influencing factors could be applied to design index insurance products for multi-factors. 3) In terms of research method, the analytical approaches of aquatic insurance are mainly classified into two categories: empirical methods and statistical analysis methods. The former includes the empirical rate method and the loss ratio method. The latter can estimate the linear relationship between influencing factors and yield by using the trend fitting analysis method. Furthermore, several scholars simulate the occurrence probability of meteorological disasters based on Weibull function model. They establish a univariate nonlinear regression model between influencing factors and yield through the Copula function to design insurance products with different meteorological indices [16–18], such as high-temperature index [19], precipitation index, continuous cloudy and rainy index [20].

Over the few decades, researchers and practitioners alike have contributed to an ongoing debate around weather index insurance’s feasibility and its merits as a tool for agricultural risk management. Most of the insurance products are designed by constructing a linear relationship between the production and environmental factors, but not all relationship between them is linear over different regions. Giacomo indicated that the Copula function can be used to measure the nonlinear relationship between variables and calculate the corresponding pure insurance rate accordingly [21]. Currently, the Copula function are usually applied in the design of index insurance products for planting crops [22], but there are few applications in the analysis of aquaculture insurance. Therefore, we attempt to use Copula function to determine the premium rate of aquaculture products. In this study, a combination of nonparametric kernel density estimation and Copula function is used to construct the probability distribution model between yield of swimming crab, SST and SSS data in typical aquaculture areas in eastern China from 2001 to 2019.

## 2 Materials and methods

### 2.1 Study area

**Fig 1:**
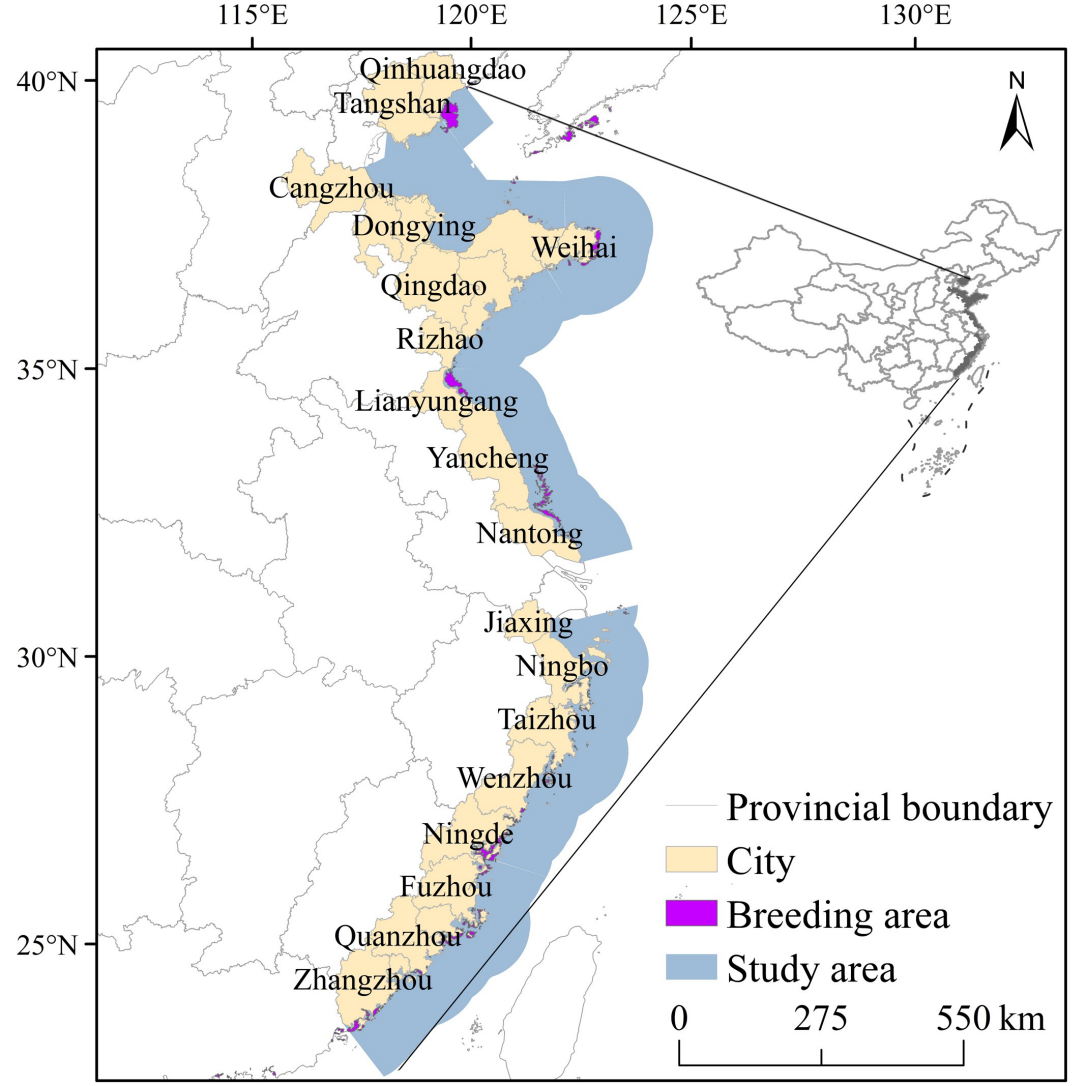
Location of the study area.

Typical areas of aquaculture in eastern China contain 24 coastal cities from Qinhuangdao to Zhangzhou (Fig 1). The swimming crab is cultivated for the water depth range at 0 to 30 meters. The SST and SSS are easily affected by solar radiation, weather conditions and internal waves. Eastern China is affected by the East Asian monsoon with large spatial and temporal variability in SST and precipitation. Various types of meteorological disasters occur every year. The SSS extreme value varies greatly in space, with the lowest value being 28.16‰ and the highest value reaching 35.71‰. The average SST in different years differs by 3.2°C. This indicate that the risk of potentially experiencing low SST varies significantly each year.

There are two stages of artificial breeding of swimming crabs, which are seedling (February to June) and crab breeding (June to February). The existing artificial seedling technology is quite mature. The breeding period of commercial crab can be subdivided into the growth period (June to November) and the breeding period (November to February). Swimming crab is a eurytherm and euryhaline species. It can survive and adapt to the water environment with water SST between 8°C and 31°C (the optimum SST is 15.5°C-26°C) and SSS of 16‰-35‰ (the optimum SSS is 26‰-32‰). If SST and SSS exceed a certain threshold level (SST<15°C and salinity>32‰), two factors can greatly affect the biological processes of swimming crab such as growth, reproduction, feeding, molting and output of the crab roe [23]. Thus, taking the coastline in China extending 100 km eastward as the study area in this study. The extreme values data of SST and SSS with three-day resolution from 2001 to 2019 are extracted resolution to investigate the effects of low SST and high SSS on the yield in the two stages of the growth period and the breeding period of commercial crab.

### 2.2 Data

The annual yield data of swimming crabs from 2001 to 2019 were collected from the Municipal Statistical Yearbooks of 24 cities. We used Simple Ocean Data Assimilation (SODA) reanalysis data sets in this study, which is jointly developed by the University of Maryland and the Texas A&M University. The SODA reanalysis is a global ocean re analysis created by assimilating observational data into an ocean general circulation model based on the POP model. It provided three-day averaged gridded variables (Sea surface salinity (SSS) (http://apdrc.soest.hawaii.edu/erddap/griddap/hawaii_soest_face_930e_5a79.html) and sea surface temperature (SST) (http://apdrc.soest.hawaii.edu/erddap/griddap/hawaii_soest_1b16_9fed_896e.html)) with a horizontal resolution of 0.1°×0.1°. The temporal and spatial resolutions are relatively coarse, but the reanalysis data can still be evaluated the relationship between yield and variables resolves most of mesoscale eddies in our study regions. The materials not included in this study are available on the OSF.

### 2.3 Methodology

#### 2.3.1 Statistical model of yield factor

The actual yield of swimming crab is affected by breeding technology, management level and natural disasters during its growth period. Therefore, it is necessary to distinguish the impact of natural and unnatural factors on yield when analyzing aquaculture insurance. Generally, the actual yield can be divided into trend yield, meteorological yield and random yield [24]. Due to the short time series selected, HP (Hodrick-Prescott) filter model can be better separate trend yield and meteorological yield (difference between actual yield and trend yield). The HP filter assumes that the yield sequence *y*_*t*_ is composed of trend term *g*_*t*_ and fluctuation term *c*_*t*_. The trending term is determined by minimizing the loss function:

$$\begin{array}{c} \min\\ {\left\{g_{t}\right\}}_{t=1}^{n} \end{array}\{\sum_{t=1}^{n}{\left(y_{t}-g_{t}\right)}^{2}\}+\{\lambda \sum_{t=1}^{n}[(g_{t+1}-g_{t})-(g_{t}-g_{t-1}){]}^{2}\}$$ (1)

Where n is the number of samples; λis smoothing parameter. According to experience, when annual data is used, the best fitting effect is *λ* = 100. Combining *y*_*t*_ = *g*_*t*_+*c*_*t*_ and Eq (1), we can be obtained the trend term as follows:

$$c_{t}=\lambda Fg_{t}$$ (2)

$$g_{t}={\left(\lambda F+I\right)}^{-1}y_{t}$$ (3)

HP filter has been widely applied in the field of yield fluctuation. The following is converted into detrended yield taking 2019 as the base period:

$$Y_{t}^{'}={\hat{Y}}_{2019}\frac{Y_{t}}{{\hat{Y}}_{t}}$$ (4)

Where *t* = 2001, …, 2019; *Y*_*t*_ is the original yield sequence; ${\hat{Y}}_{t}$ is the trend yield in year *t*.

#### 2.3.2 Nonparametric kernel density estimation

Unlike parametric methods, the nonparametric kernel density estimation does not need to make definite assumptions about the distribution of the data. It relies on the characteristics of data to obtain its distribution. The kernel function and bandwidth can achieve the fitting of the probability density. It can not only be widely used, but also makes the fitted probability destiny functions (PDF) closer to the real information.

Respectively, taking the variables yield, SST, and SSS data to calculate the volatility of the variables, the probability density function f(x) can be expressed as [25]:

$$f_{h}(x)=\frac{1}{nh}\sum_{i=1}^{n}K(\frac{x-x_{i}}{h})$$ (5)

Where *K(x)* is the kernel function; *n* is the sample size; *h* is the window width; It is necessary to satisfy *K(x)*≥0, $\int_{-\infty }^{+\infty }K(x)dx=1$; The commonly used *K(x)* is a Gaussian kernel function, which serves to calculate the premium rate of swimming crab. The expression is:

$$K_{h}(x)=\frac{1}{\sqrt{2}\pi }\exp(-\frac{x^{2}}{2})$$ (6)

According to Silverman’s rule of thumb, the optimal bandwidth of the Gaussion kernel function is:

$$h=0.9\times \min\{\mathrm{standard}\;\mathrm{deviation},\;\frac{\mathrm{interquartile}\;\mathrm{range}}{1.34}\}\times n^{-\frac{1}{5}}$$ (7)

#### 2.3.3 Construction joint probability model for yield and SST or SSS

Copula concept is proposed as a function which can combines one-dimensional marginal distribution functions to form a multivariate distribution function between two or more random variables by Sklar in 1959 [26–28]. The Sklars theorem propose: If the binary the joint distribution function of random vector *X*, *Y* is H and has continuous marginal distribution functions are *F*, *G*, then there is a Copula function *C*(*·*):

$$C(u,v)=H(F^{-1}(u),G^{-1}(v))$$ (8)

Where *X* and *Y* are SST and SSS factors respectively; *F*^−1^ and *G*^−1^ mean the generalized inverse of *F* and *G*, that is, $F^{-1}(u)=\sup_{z}\{F(z)\leq u\}$ and $G^{-1}(v)=\sup_{z}\{G(z)\leq v\}$; *F* and *G* are marginal distribution functions of yield and SST and SSS factors respectively; *C* is the joint distribution of marginal distribution on [0,1]; then the density function of the Copula function expressed as:

$$c(F(x),G(y))=\frac{\partial }{\partial x\partial y}C(F(x),G(y))$$ (9)

If the derivative exists, the joint density of *X* and *Y* is given by f(x,y)=c(F(x),G(y))f(x)g(y), where f and g are the probability density functions of *F* and *G*.

Currently, the Copula function is applicable for pricing crop income insurance in the field of insurance finance. The nonlinear dependence relation between variables can be calculated by constructing joint distribution among variables. There are four common types of Copula functions selected in this paper including Normal Copula, Gumbel Copula, Frank Copula, and Clayton Copula. The specific structure of the Copula functions is uniformly expressed as in:

$$C(u_{1},u_{2},\ldots u_{n})=\varphi^{-1}(\varphi (u_{1})+\ldots +\varphi (u_{n}))$$ (10)

Where *φ*: [0,1]→[0,∞], is the generator of the Copula function, and is a continuous strictly decreasing function such that *φ*(1) = 0; *φ*^−1^ mean the inverse of *φ*, is a continuous and non-increasing function defined on [0,∞], such that $\varphi^{-1}(t)=\{\begin{array}{l} \varphi^{-1}(t)\;0\leq t\leq \varphi (0),\\ \;0\;\varphi (0)<t\leq \infty . \end{array}$ The generators and equations of the Copula function are shown in Table 1.

**Table 1:** Copula function equation and generator.

Copula function	Equation	Generator
Normal Copula	$C(u,v)=\frac{1}{2\pi \sqrt{1-\theta^{2}}}\int_{-\infty }^{\varphi^{-1}(u)}\int_{-\infty }^{\varphi^{-1}(v)}\exp[\frac{-(s^{2}-2\theta st+t^{2})}{2(1-\theta^{2})}]dsdt$	Standard normal distribution
Gumbel Copula	$C(u,v)=\exp[-{\left[{\left(-\ln u\right)}^{\theta }+{\left(-\ln v\right)}^{\theta }\right]}^{1/\theta }]$	${\left(-\ln x\right)}^{\theta },\theta \in [1,+\infty ]$
Frank Copula	$C(u,v)=(-1/\theta )\ln[1+(e^{-\theta u}-1)(e^{-\theta v}-1)/(e^{-\theta }-1)]$	$-\ln[(e^{-\theta x}-1)/(e^{-x}-1)],\theta \in (-\infty ,\infty )\backslash \{0\}$
Clayton Copula	$C(u,v)=\max[{\left(u^{-\theta }+v^{-\theta }-1\right)}^{-1/\theta },0]$	$\left(1/\theta )(x^{-\theta -1}),\theta \in [-1,+\infty ]\backslash \{0\right\}$

The joint distribution of yield, SST and SSS parameters are separately estimated by the maximum likelihood method. MATLAB software is used to calculate the square Euclidean distances between the four Copula functions and the empirical Copula functions, respectively. The optimal copula adopts the principle of least squared Euclidean distance from the empirical copula. Copula function has correlation information between yield and SSS, yield and SST, and is embodied by Kendall-*τ* correlation coefficient.

#### 2.3.4 Pure premium rate

Monte Carlo simulation model is used to separately determine premium rates for the yield and SSS, yield and SST. Firstly, the Copula function form and its parameters calculated in the previous step are sampled 10,000 times by Monte Carlo, and a random sequence *u*, *v* conforming to the [0,1] distribution is simulated and generated. Secondly, the inverse function of the marginal distribution is calculated by the spline interpolation method. Finally, the yield, SST and SSS data are obtained through reduction processing, and the newly generated yield is taken as the yield sample data under such SST or SSS conditions, and then substituted into the following premium calculation formula to determine the premium rate [29]:

$$Expectdloss(y)=prob(y<\alpha \bar{y})[\alpha \bar{y}-E(y|y<\alpha \bar{y})]$$ (11)

$$r=\frac{Expectdloss(y)}{ay}$$ (12)

Where *α* is the coverage level, *α*∈[0,1]; $\bar{y}$ is the expected yield level, substitute with the average long-term yield of swimming crabs.

## 3 Results

### 3.1 Data preprocessing

A stationarity test is applied to the original data and the results show that the original yield, SST and SSS series of most cities were non-stationary. Then, the non-stationary series are detrended to ensure the accuracy of the simulation results. The non-stationary original series of yield, SST and SSS are decomposed by the HP filter model. Moreover, the yield is converted into the yield data with 2019 as the base period. The ADF stationarity test is performed again on the detrended yield, SSS and SST series of 24 cities (Table 2). Because the yield, SSS and SST data have different dimensions, the normalization method is required for dimensionless processing.

**Table 2:** Test results of sequence stationarity after detrending in 24 cities.

City	Yield ADF test	SSS ADF test	SST ADF test	City	Yield ADF test	SSS ADF test	SST ADF test
Qinhuangdao	-3.127*	-3.797*	-3.234*	Nantong	-4.209**	-3.665*	-3.161*
Tangshan	-3.503*	-3.883*	-3.102*	Jiaxing	-4.087**	-3.851*	-3.076*
Cangzhou	-5.272**	-3.392*	-3.135**	Ningbo	-3.879**	-4.202**	-3.244*
Binzhou	-4.311**	-3.176*	-3.110*	Zhoushan	-3.060*	-4.615**	-3.245*
Dongying	-3.642*	-3.214*	-3.219*	Taizhou	-3.561*	-5.121**	-3.593*
Weifang	-4.504**	-3.154*	-3.071*	Wenzhou	-3.284*	-3.247*	-3.849*
Yantai	-3.104*	-3.105*	-3.146*	Ningde	-3.565*	-3.194*	-4.157**
Weihai	-3.272*	-3.298*	-3.357*	Fuzhou	-3.776*	-3.130*	-4.180**
Qingdao	-4.256**	-3.513*	-3.301*	Putian	-3.162*	-3.152*	-4.553**
Rizhao	-3.149*	-3.118*	-3.189*	Quanzhou	-3.841*	-3.178*	-3.349*
Lianyungang	-3.714*	-5.992**	-3.207*	Xiamen	-3.709*	-3.156*	-6.778**
Yancheng	-4.134**	-4.099**	-3.119*	Zhangzhou	-3.136*	-3.313*	-5.605**

Note:

"**" means that the ADF test reaches the 0.01 significance level

"*" means that the ADF test reaches the 0.05 significance level, where 1% critical value is -3.872, and 5% critical value is -3.046.

**Fig 2:**
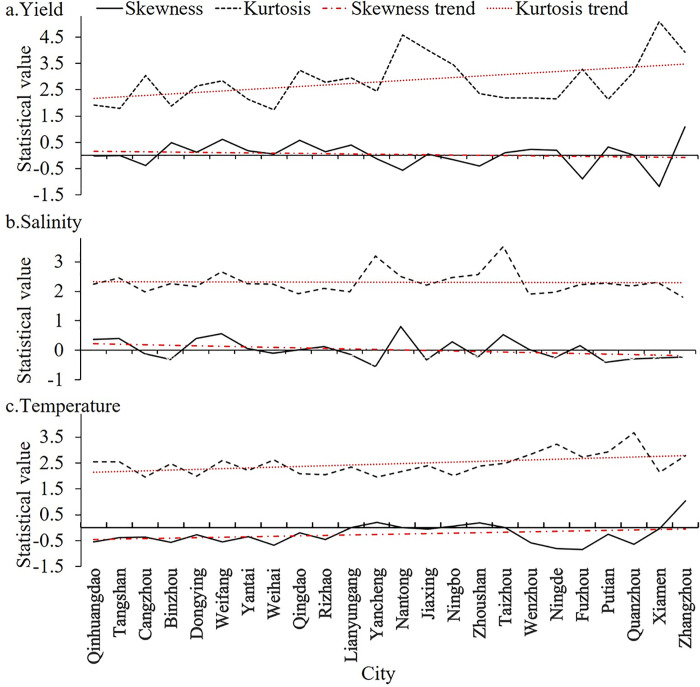
Descriptive statistics of yield, SST and SSS after detrending in 24 cities.

There are significant differences in skewness and kurtosis of yield after detrending in 24 cities (Fig 2). The kurtosis curve of yield shows a fluctuating upward trend with decreasing latitude (Fig 2A). The yield is mostly in the form of "low peaks and thin tails" in the cities north of Yancheng, and mainly in the form of "sharp peaks and thick tails" in the cities south of Yancheng. The skewness and kurtosis of SSS and SST show similar spatial variation trends in the study area. The skewness and kurtosis of SSS show a decreasing trend (Fig 2B), while the skewness and kurtosis of SST show an increasing trend (Fig 2C). Since kurtosis values of SST and SSS in cities are both less than 3, except for Yancheng, Taizhou, Ningde, and Quanzhou, indicating that the SST and SSS series have a "low peak and thin tail" shape on the whole. The morphology of the analyzed data series is used to compare with the fitted kernel density function plot to test whether the nonparametric estimation method fits well.

### 3.2 Construction joint probability model

#### 3.2.1 Determine the marginal distribution

**Fig 3:**
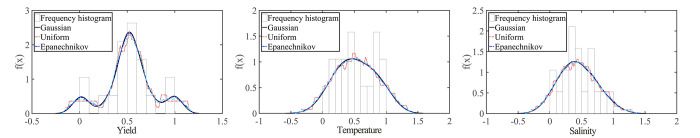
Estimation of Gaussian kernel density for yield, SST and SSS of swimming crab in Ningbo city.

The parametric estimation and the nonparametric kernel density estimation method are used to simulate the probability distributions trend yield, SST and SSS, and the results produced by nonparametric method can be more accurate than the results given by parametric estimation. In this study, four different kernel functions such as Uniform, Triangular, Epanechnikov and Gaussian are used to fit the marginal distribution of variables. Taking Ningbo as an example, the kernel density estimation results are analyzed. According to the Silverman’s rule-of-thumb, the window widths of yield, SST and SSS of swimming crab Ningbo are calculated as 0.084, 0.173 and 0.195, respectively. The Gaussian kernel function estimates the distribution of yield, SST and SSS pretty accurately (Fig 3), which is consistent with the results of descriptive statistical analysis. Similarly, the Gaussian kernel function also has the best fitting in other cities. Therefore, the premium rate of swimming crabs is calculated by the marginal distribution function fitted by the Gaussian kernel function.

#### 3.2.2 Copula parameter estimation

The Kendall-*τ* correlation coefficient is used to calculate the correlation between yield and its influencing factors. The optimal Copula function correlation coefficient between yield and SSS is negative in 7 cities such as Cangzhou (Table 3), which indicates that a large increase in SSS may lead to yield reduction. On the contrary, the Kendall-*τ* correlation coefficient of 9 cities such as Qinhuangdao is greater than 0, showing a trend of increasing production, while the Kendall-*τ* correlation coefficient of 8 cities such as Dongying is close to 0. It indicates that the relationship between the yield and SSS is different in space. We also examine the correlation between SST and yields and found that there is a weak negative correlation except for Ningbo, Wenzhou and Quanzhou, and all other cities are positive correlations. It is shown that the decrease in SST led to the reduction in yields.

**Table 3:** Copula function estimation results in 24 cities.

	Yield and SSS				Yield and SST			
City	Copula function form	parameter(θ)	Kendall-*τ*	Squared Euclidean distance	Copula function form	parameter(θ)	Kendall-*τ*	Squared Euclidean distance
Qinhuangdao	Clayton	0.453	0.185	0.012	Clayton	0.185	0.085	0.026
Tangshan	Clayton	0.222	0.100	0.016	Clayton	0.456	0.186	0.038
Cangzhou	Frank	-2.512	-0.263	0.033	Gaussian	0.157	0.101	0.016
Binzhou	Frank	1.305	0.143	0.021	Clayton	0.099	0.047	0.011
Dongying	Frank	0.201	0.022	0.023	Clayton	0.277	0.121	0.019
Weifang	Frank	0.892	0.098	0.015	Gaussian	0.130	0.083	0.014
Yantai	Gaussian	0.263	0.169	0.015	Frank	0.034	0.004	0.014
Weihai	Gaussian	-0.174	-0.112	0.016	Clayton	0.795	0.284	0.011
Qingdao	Gumbel	1.181	0.153	0.006	Clayton	0.210	0.095	0.022
Rizhao	Clayton	0.628	0.239	0.014	Frank	0.809	0.089	0.014
Lianyungang	Gaussian	-0.034	-0.022	0.042	Clayton	0.437	0.179	0.013
Yancheng	Gumbel	1.050	0.047	0.021	Clayton	0.295	0.129	0.009
Nantong	Gaussian	0.155	0.099	0.008	Clayton	0.823	0.292	0.019
Jiaxing	Frank	-1.016	-0.112	0.030	Frank	0.638	0.071	0.021
Ningbo	Gumbel	1.075	0.070	0.011	Frank	-0.221	-0.025	0.013
Zhoushan	Frank	-0.822	-0.091	0.017	Clayton	0.112	0.053	0.013
Taizhou	Frank	-1.973	-0.211	0.015	Clayton	0.468	0.190	0.011
Wenzhou	Gaussian	-0.344	-0.223	0.024	Gaussian	-0.179	-0.115	0.016
Ningde	Frank	-0.890	-0.098	0.023	Frank	0.368	0.041	0.020
Fuzhou	Frank	0.450	0.050	0.011	Clayton	0.098	0.047	0.009
Putian	Clayton	0.000	0.000	0.012	Clayton	0.194	0.088	0.015
Quanzhou	Gaussian	0.012	0.008	0.023	Gaussian	-0.235	-0.151	0.027
Xiamen	Clayton	0.000	0.000	0.011	Clayton	0.047	0.023	0.022
Zhangzhou	Gaussian	0.002	0.002	0.009	Gumbel	1.000	0.000	0.014

### 3.3 Index insurance premium rates

The pure premium rate can be calculated using Eq (6,7) for a given level of coverage (from 70 to 100% with an interval of 5%). The results are showed in Table 4. The pure premium rate increases rapidly with the increasing in level of coverage. Within the range of 70%-100% of the coverage level, premium rates of SST and SSS index insurance in the same city fluctuates by 10%. When the coverage level is higher, the increase in the variation of pure premium rate is larger. Especially when the coverage level is 100%, the pure premium rate increase span is between 1.6% and 2.04%. The pure premium rates of swimming crabs in different cities vary greatly. It can be seen that the premium rates of Taizhou and 8 cities south of Taizhou are lower than the 16 cities north of Taizhou. When the coverage level is 100%, the premium rates is between 6% and 22% for most cities in north of Taizhou and less than 5.6% for cities in the south of Taizhou. There will be no expected loss when the coverage level is lower than 85% for cities in the south of Taizhou. The premium rates for different provinces are also examined, and find that the premium rates of swimming crabs are less in southern Zhejiang and Fujian, higher in Jiangsu, and much higher in Hebei, Shandong, and northern Zhejiang.

**Table 4:** Results of determination of pure insurance rates in 24 cities including Qinhuangdao (%).

Assurance leve	Qinhuangdao		Tangshan		Cangzhou		Binzhou		Dongying		Weifang		Yantai		Weihai	
	SSS	Tempe-rature	SSS	Tempe-rature	SSS	Tempe-rature	SSS	Tempe-rature	SSS	Tempe-rature	SSS	Tempe-rature	SSS	Tempe-rature	SSS	Tempe-rature
100%	11.44	11.76	10.59	11.26	11.66	12.06	21.61	22	9.77	10.08	8.89	9.16	7.83	8.05	8.99	9.64
95%	9.55	9.89	8.67	9.37	9.78	10.18	20.19	20.58	7.82	8.14	6.91	7.17	5.82	6.04	7.02	7.7
90%	7.76	8.1	6.87	7.59	7.99	8.38	18.78	19.18	6.01	6.33	5.1	5.36	4.03	4.23	5.21	5.9
85%	6.08	6.41	5.2	5.92	6.31	6.67	17.37	17.78	4.36	4.67	3.5	3.74	2.5	2.67	3.6	4.28
80%	4.53	4.86	3.69	4.39	4.75	5.09	15.96	16.39	2.91	3.21	2.13	2.34	1.28	1.43	2.22	2.87
75%	3.14	3.45	2.37	3.04	3.35	3.65	14.56	14.99	1.69	1.96	1.04	1.23	0.42	0.56	1.12	1.67
70%	1.94	2.23	1.29	1.89	2.12	2.4	13.16	13.59	0.75	0.98	0.31	0.45	0.02	0.12	0.35	0.77
Assurance level	Qingdao		Rizhao		Lianyungang		Yancheng		Nantong		Jiaxing		Ningbo		Zhoushan	
	SSS	Tempe-rature	SSS	Tempe-rature	SSS	Tempe-rature	SSS	Tempe-rature	SSS	Tempe-rature	SSS	Tempe-rature	SSS	Tempe-rature	SSS	Tempe-rature
100%	14.59	14.81	15	15.29	6	6.51	6.09	6.46	7.85	8.55	17.46	17.69	11.24	11.31	6.62	6.93
95%	12.84	13.07	13.27	13.54	3.96	4.49	4.05	4.42	5.84	6.58	15.84	16.06	9.35	9.4	4.59	4.91
90%	11.13	11.37	11.57	11.85	2.27	2.79	2.35	2.72	4.05	4.8	14.24	14.46	7.55	7.58	2.85	3.16
85%	9.47	9.72	9.93	10.2	0.99	1.46	1.06	1.4	2.52	3.25	12.66	12.87	5.87	5.89	1.47	1.76
80%	7.87	8.14	8.33	8.58	0.21	0.57	0.24	0.52	1.3	1.96	11.11	11.31	4.33	4.35	0.5	0.75
75%	6.35	6.61	6.81	7.04	0	0.1	0	0.09	0.44	0.97	9.59	9.78	2.95	2.98	0.03	0.18
70%	4.92	5.17	5.36	5.57	0	0	0	0	0.02	0.31	8.11	8.29	1.78	1.8	0	0.01
Assurance level	Taizhou		Wenzhou		Ningde		Fuzhou		Putian		Quanzhou		Xiamen		Zhangzhou	
	SSS	Tempe-rature	SSS	Tempe-rature	SSS	Tempe-rature	SSS	Tempe-rature	SSS	Tempe-rature	SSS	Tempe-rature	SSS	Tempe-rature	SSS	Tempe-rature
100%	3.03	4.4	3.77	3.91	3.71	4.03	3.8	3.89	3.93	2.88	5.6	5.51	4.4	4.42	4.48	4.47
95%	1.1	2.47	1.77	1.91	1.72	2.03	1.8	1.91	1.92	1	3.56	3.47	2.37	2.39	2.45	2.45
90%	0.1	1.14	0.47	0.66	0.44	0.72	0.49	0.59	0.58	0.11	1.91	1.83	0.91	0.92	0.97	0.97
85%	0	0.33	0	0.13	0	0.11	0	0.04	0	0	0.72	0.67	0.11	0.12	0.14	0.14
80%	0	0	0	0	0	0	0	0	0	0	0.08	0.07	0	0	0	0

The rates observed in this study is similar to the rates from previous studies which is determined by different other methods (Table 5). The correlation coefficient between the rate of the parametric distribution fitting method and the rate that based on the coupling of the Copula function and the nonparametric kernel density estimation is 0.957, and the correlation coefficient with the kernel density estimation results of other scholars is 0.946, both of which pass the 0.05 significance test. It can be stated that the method used in this study can also be used as a rate determination method. Since the 20% relative deductible rate specified in actual insurance products is not considered in this paper, the rate results are higher than those of other scholars. In addition, it is found that the two rates are similar by comparing the rate of the swimming crab precipitation insurance index product actual operating in Xiangshan County with the pure premium rate of the Ningbo swimming crab SST and SSS index insurance calculated in this paper. Therefore, it is of certain practical significance to take advantage of the Copula function to combine production data with SST and SSS data to determine the pure premium rate.

**Table 5:** Comparison of rate results of different methods.

Methods	Hebei	Shandong	Jiangsu	Zhejiang	Fujian
Empirical rate [30]	8.43	5.94	0	3.11	0
Parametric distribution fitting [30]	9.78	7.77	0.39	2.57	0
Kernel density estimation [30]	10.10	8.32	0.55	3.66	0.08
Copula function coupled with kernel density estimation	11.23	12.38	6.64	8.42	4.32

## 4 Discussion

There is little literature regarding the determination of the pure premium rate of swimming crab in typical aquacultural area from China’s aquaculture insurance for pilots. Currently, there are several ways to verify the rationality of the pure premium rate of swimming crabs in the study area based on nonparametric kernel density estimation and Copula function calculation:

### 4.1 Discussion based on the methods

Currently, the empirical rate method is the most commonly method used to determine the premium rate of aquaculture [31] It is a method of approximately replacing the premium rate with the average loss rate of the calendar year. The nonparametric kernel density estimation method takes the actual recorded data as a sample and does not need to assume a specific distribution form to estimate the loss probability, thus the results of this method will be more accurate and reliable. In theory, the rate calculated by the nonparametric kernel density estimation method can be regarded as an actuarially fair rate. We compare the difference between the empirical rate method and the pure premium rate determined by several other methods and find that the empirical rate is less than Actuarial fair rates, which also explains the high rate of mariculture insurance projects [32].

### 4.2 Discussion based on the large spatial variability

The provinces with higher pure premium rates in the study area are Hebei, Shandong and Zhejiang, while with lower pure premium rates is Fujian. These features are based on the pure premium rate in the study area calculated by the Copula function, which is similar to the results of the risk zoning of crustaceans in the Chinese aquaculture study by Gao et al. [32]. However, when comparing the changes in the pure premium rates of aquaculture with the city as a unit, it shows that the pure premium rates of swimming crabs vary in different cities.

It is clear that the significant differences between cities in terms of the aquaculture cultured area, the frequency and intensity of disasters can lead to boost differences in the risk of loss between cities. Thus, the rates under the city scale is higher than those under the provincial scale, and the variation range of pure premium rates in different cities is between 4% and 22% [33]. It can be seen that the current rate standards adopted by insurance institutions are prone to high compensation and high expenses. In other words, applying the same rate on a large scale may cause unfairness [34]. It is suggested that insurance institutions and local governments should actively promote the risk assessment work in coastal county-level areas to lays the foundation for more accurate premium rates.

### 4.3 Discussion based on research data

The premium rates for existing research aquaculture insurance products are as high as 8% or even higher [30], but there are large differences in the premium rates of swimming crabs in different regions. Since there is no standard production record, insurance companies may only obtain sample data on losses of mariculture households in the last 3–5 years to calculate premium rates. Therefore, even the same method is used to calculate different pure rates at different sample sizes [35]. Some short time series are not enough to reflect the actual risk, while some long time series are susceptible to changes in production risk over time in practice, so it is more appropriate to take a sample time series of fewer than 20 years when determining rates.

There are also significant differences in the premium rate of different species. The premium rate of shrimp and scallop can be as high as 21%, and the premium rate of fish is even higher. The reason may be related to the adaptability of cultured species to environmental changes. In addition, the scale of aquaculture also has an impact on insurance rates. Because the area at risk in the event of a disaster are becoming larger as the scale of aquaculture increased. Therefore, the government should adopt different subsidy policies to deal with the situation, which there are large differences in the pure premium rates of swimming crabs in various regions. It is necessary to develop products that meet the needs of the local market according to local conditions. It can reflect that government work is more scientific and fairness.

The following is a summary of the outlook for index insurance based on the current situation of the development of domestic index aquaculture insurance: Local governments should strengthen cooperation with enterprises and universities, establish a comprehensive marine aquaculture disaster database and insurance compensation database, and enrich the city and county-level data. Because different cities and counties have different degrees of aquaculture risks. Aquaculture households are difficult to obtain insurance compensation data, and information acquisition is not timely. If people can design more scientific and reasonable rate insurance products, it will help to promote the stable development of aquaculture. Moreover, it is necessary to strengthen the cross-integration of multidisciplinary knowledge and the innovative application of digital technology. It can combine satellite remote sensing, geographic information, artificial intelligence processing technology, and monitored marine environmental data to improve the accuracy of aquatic insurance judgment and design more sophisticated insurance products. At the same time, it is also significant to enrich the ways of inspection and damage determination and reduce the investment of human and material resources.

There are many natural factors affecting the yield of swimming crabs. It is not comprehensive enough to only consider the effects of SST and SSS on the yield of swimming crabs. In future research, we should comprehensively consider the variety of influencing factors and enlarge the time series length of the yield of swimming crabs and SST and SSS data to design corresponding index insurance to improve the accuracy of determining the index insurance of swimming crabs.

## 5 Conclusions

Quantifying the relationship between influencing factors and aquatic yield that is critical to the design of index insurance. We combine nonparametric kernel density estimation with copula function to establish marginal distribution and joint distribution of yield with SST and SSS in the study area from 2001 to 2019. The distribution is sampled using Monte Carlo simulations to obtain a large amount of yield, SST, and SSS data. On this basis, the pure premium rate of swimming crabs in 24 cities is determined under the coverage level of 70%-100%. There are three majorly results as follows:

1. SSS increase in the northern part of the study area made a large contribution to the increase of the yield, while the yield in the southern region is not significantly affected by SSS. The yield of swimming crabs in most cities increases with the increase of SST.
2. The pure premium rate of swimming crabs in each city area varies by less than 10% when the coverage level was between 70% and 100%. Furthermore, the change amplitude of pure premium rate for swimming crabs increase with the higher the level of coverage.
3. The pure premium rates of swimming crab vary significantly across 24 cities. The pure premium rates of swimming crab are low in 8 cities south of Taizhou, suggesting that both temperature and salinity pose less risk to yields in these cities. However, there are enormous differences in pure premium rates for swimming crabs between 16 cities north of Taizhou. The premium rate is between 6% and 22%.

## Supporting information

S1 File (ZIP)
