# Supplementary material for: Research on the insurance of swimming crab temperature and salinity index insurance based on Copula function
Source: PLoS One. 2022 Aug 15;17(8):e0272940. doi: 10.1371/journal.pone.0272940 (PMC9377581; doi:10.1371/journal.pone.0272940)
Supplement: Peer review history

# Peer review: Decision Letter 0

**DOI:** 10.1371/journal.pone.0272940.r001

**Authors:** Mine Perçin Olgunoglu

1 Aug 2022

Research on the insurance of swimming crab temperature and salinity index insurance based on Copula function

PONE-D-22-18070

Dear Dr. Gao

We’re pleased to inform you that your manuscript has been judged scientifically suitable for publication and will be formally accepted for publication once it meets all outstanding technical requirements.

Within one week, you’ll receive an e-mail detailing the required amendments. When these have been addressed, you’ll receive a formal acceptance letter and your manuscript will be scheduled for publication.

An invoice for payment will follow shortly after the formal acceptance. To ensure an efficient process, please log into Editorial Manager at http://www.editorialmanager.com/pone/, click the 'Update My Information' link at the top of the page, and double check that your user information is up-to-date. If you have any billing related questions, please contact our Author Billing department directly at authorbilling@plos.org.

If your institution or institutions have a press office, please notify them about your upcoming paper to help maximize its impact. If they’ll be preparing press materials, please inform our press team as soon as possible -- no later than 48 hours after receiving the formal acceptance. Your manuscript will remain under strict press embargo until 2 pm Eastern Time on the date of publication. For more information, please contact onepress@plos.org.

Kind regards,

Mine Perçin Olgunoglu

Academic Editor

PLOS ONE

Journal requirements:

1. PLOS ONE publication criteria requires that research must be described in enough detail to allow readers to fully replicate the study (https://journals.plos.org/plosone/s/criteria-for-publication#loc-3), and that all data underlying the findings described in their manuscript must be fully available (https://journals.plos.org/plosone/s/criteria-for-publication#loc-7). Please amend your Methods section to provide the URL for the specific data sets used and please provide the datasets as a supplemental file.

2. PLOS requires an ORCID iD for the corresponding author in Editorial Manager on papers submitted after December 6th, 2016. Please ensure that you have an ORCID iD and that it is validated in Editorial Manager. To do this, go to ‘Update my Information’ (in the upper left-hand corner of the main menu), and click on the Fetch/Validate link next to the ORCID field. This will take you to the ORCID site and allow you to create a new iD or authenticate a pre-existing iD in Editorial Manager. Please see the following video for instructions on linking an ORCID iD to your Editorial Manager account: https://www.youtube.com/watch?v=_xcclfuvtxQ

Additional Editor Comments:

The work contains important information.

Reviewers' comments:

# Editor report: Acceptance letter

**DOI:** 10.1371/journal.pone.0272940.r002

**Authors:** Mine Perçin Olgunoglu

5 Aug 2022

PONE-D-22-18070 

Research on the insurance of swimming crab temperature and salinity index insurance based on Copula function 

Dear Dr. Gao:

I'm pleased to inform you that your manuscript has been deemed suitable for publication in PLOS ONE. Congratulations! Your manuscript is now with our production department. 

If your institution or institutions have a press office, please let them know about your upcoming paper now to help maximize its impact. If they'll be preparing press materials, please inform our press team within the next 48 hours. Your manuscript will remain under strict press embargo until 2 pm Eastern Time on the date of publication. For more information please contact onepress@plos.org.

If we can help with anything else, please email us at plosone@plos.org. 

Thank you for submitting your work to PLOS ONE and supporting open access. 

Kind regards, 

PLOS ONE Editorial Office Staff

on behalf of

Dr. Mine Perçin Olgunoglu 

Academic Editor

PLOS ONE
